# Improving crop disease resistance: lessons from research on *Arabidopsis* and tomato

**DOI:** 10.3389/fpls.2014.00671

**Published:** 2014-12-03

**Authors:** Sophie J. M. Piquerez, Sarah E. Harvey, Jim L. Beynon, Vardis Ntoukakis

**Affiliations:** School of Life Sciences, University of WarwickCoventry, UK

**Keywords:** *Arabidopsis*, tomato, model, crop engineering, disease resistance, food security

## Abstract

One of the great challenges for food security in the 21st century is to improve yield stability through the development of disease-resistant crops. Crop research is often hindered by the lack of molecular tools, growth logistics, generation time and detailed genetic annotations, hence the power of model plant species. Our knowledge of plant immunity today has been largely shaped by the use of models, specifically through the use of mutants. We examine the importance of *Arabidopsis* and tomato as models in the study of plant immunity and how they help us in revealing a detailed and deep understanding of the various layers contributing to the immune system. Here we describe examples of how knowledge from models can be transferred to economically important crops resulting in new tools to enable and accelerate classical plant breeding. We will also discuss how models, and specifically transcriptomics and effectoromics approaches, have contributed to the identification of core components of the defense response which will be key to future engineering of durable and sustainable disease resistance in plants.

## INTRODUCTION

Plants are exposed to a wide-range of pests and pathogens, encompassing bacteria, fungi, oomycetes, viruses, nematodes, and insects but only in specific interactions does this result in disease. However, an average of 26% of the worldwide crop production is lost each year due to pre-harvest pests and pathogens (Oerke, 2006). Increasing human populations, loss of agricultural land due to climate change, erosion and lack of water require that we reduce production losses such as those caused by pathogens as much as possible (Bebber et al., 2013). The four major staples produced worldwide, rice (which feeds more than half the world population), wheat, maize and banana are constantly threatened by damaging emerging infectious diseases in both developing and developed countries (Fisher et al., 2012). Serious biological threats to food security include *Puccinia graminis* f. sp. *tritici* (the causal agent of wheat stem rust), a highly virulent strain of which (Ug99) emerged in 1998 in East Africa. This strain overcomes the resistance genes used in crops to combat stem rust, and has spread dramatically on the African continent and to Asia (Ayliffe et al., 2008). Another serious threat, the oomycete pathogen *Phytophthora infestans* is the causative agent of late blight of potato and tomato, resulting in the deaths of 1.25 million people during the Irish potato famine of 1845 and remaining a contemporary problem (Haverkort et al., 2008; Pennisi, 2010). In addition, Black Sigatoka caused by *Mycosphaerella fijiensis* and Panama disease caused by *Fusarium oxysporum* f. sp. *cubensis* threaten banana fields, a major staple food in developing countries (Butler, 2013) and soybean production worldwide is constrained by cyst nematodes and rust caused by the fungus *Phakopsora pachyrhizi* (Carmona et al., 2005; Pivonia et al., 2005). Compounded by poleward movement of pathogens due to climate change (Bebber et al., 2013), there are numerous examples of emerging disease that may develop into uncontrollable epidemics and jeopardize food security if countermeasures are not deployed.

In the absence of genetic resistance in crops, food production heavily relies on chemical control of pathogens. Despite their effectiveness, copper based chemicals have detrimental environmental consequences, building up in the soil and appearing in water leaching from fields creating risks to the wider environment. Modern synthetic chemicals usually have reduced environmental toxicity, however, they are expensive and only available to advanced agricultural production systems. As with antibiotics, discovery of new chemistry is difficult and extensive use of current agents may result in selection of pathogen strains tolerant to pesticides (Marco and Stall, 1983; Childers et al., 2014; Hahn, 2014). Reducing the dependence of food production on chemical control is a key goal of plant pathology research. Hence, to ensure sustainable food security, we need to engineer long-lasting and broad-spectrum disease resistance in crops. One of the major goals of plant research in the 21st century is to increase our understanding of the plant immune system and unravel how this is manipulated by pathogens, in order to engineer transgenic crops with both durable resistance against pathogens and increased yields (Dangl et al., 2013).

In this review we discuss how advances in model systems have been instrumental in the emergence of new ways to manipulate the host defense responses (**Table 1**). Work from *Arabidopsis* and tomato has allowed us to build comprehensive models of the plant immune system and elucidate the mechanism used by pathogens to suppress its effectiveness (Jones and Dangl, 2006). We have now reached the stage where it is possible to propose holistic ways to alter crop genetics with the aim of engineering low cost durable resistance mechanisms in crop plants.

**Table 1:** Well-described examples of transfer of research from models to crops.

	Pathogen	Model plants	Application in crops
*NLR* genes	Broad range of pathogens	*R* gene discovery, cloning and understanding	More efficient *R* gene cloning and intra- and interspecies transfer
		*Arabidopsis RPS4/RRS1* (Narusaka et al., 2009)	Transfer of *RPS4/RRS1* to Brassicas, cucumber (Narusaka et al., 2013a)
Transfer of *PRRs* across species	Broad range of phytopathogenic bacteria	*Arabidopsis EFR* tested in tomato (Lacombe et al., 2010)	Transfer of *EFR* into crops such as potato, lettuce, apple, citrus^a^
		Tomato *Ve1* tested in *Arabidopsis* (Fradin et al., 2011)	
Host-induced gene silencing (HIGS)	Crown gall; *Agrobacterium tumefaciens*	Proof of concept: silencing of *iaaM* and *ipt* in *Arabidopsis* and tomato (Escobar et al., 2001)	Silencing of *iaaM*, *ipt*, *Pv010* in walnut roots (Walawage et al., 2013)
	Root knot nematodes; *Meloidogyne* spp.	Silencing of *Arabidopsis 16D10* (Huang et al., 2006)	Silencing of *16D10* in grape (Yang et al., 2013b)
	*Fusarium* head blight; *Fusarium graminearum*	Silencing of *Arabidopsis CYP51* (Koch et al., 2013)	Silencing of *CYP51* in barley (Koch et al., 2013)
Non-host resistance	Asian soybean rust; *Phakopsora pachyrhizi*	Identification of *BRT1* as a component of non-host resistance (Langenbach et al., 2013)	*BRT1* transferred into soybean (Conrath et al., 2013)
Transgenerational systemic acquired resistance (SAR)	*Pseudomonas syringae*, *Hyaloperonospora arabidopsidis* and *Rhynchosporium commune*	Heritable resistance in *Arabidopsis* due to pathogen (Luna et al., 2012) or chemical treatment (Slaughter et al., 2012)	Heritable resistance in barley conferred upon pathogen and chemical treatment (Walters and Paterson, 2012); heritable resistance to wheat streak mosaic virus in wheat (Seifers et al., 2014)

*^a^http://2blades.org*

## *Arabidopsis* AND TOMATO AS MODELS IN PLANT–PATHOGEN INTERACTIONS

**FIGURE 1:**
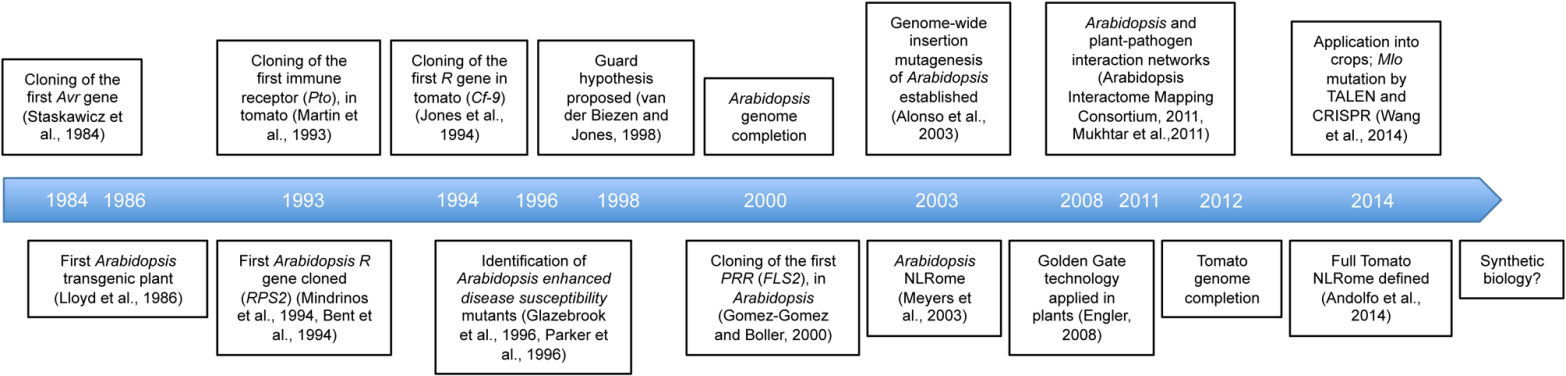
**Milestones associated with the use of *Arabidopsis* and tomato as research models**.

Tomato (*Solanum lycopersicum*) belongs to the Solanaceae family, which encompasses many important crops such as potato, pepper, and eggplant. It is the second most important vegetable crop worldwide, having reached a production of more than 160 million tones FAOSTAT (2012). Historically, tomato was a reference species and a model not only for fleshy, berry-type fruit development and ripening but also for plant–pathogen interactions (Giovannoni, 2001, 2004; Takahashi et al., 2005; Arie et al., 2007). Tomato plants are infected by a plethora of diseases (Jones et al., 1991) and given that tomato was easily transformable, had transposons and a defined pathogen genetics with *Cladosporium fulvum*, it stood as an excellent model of choice to unravel disease resistance. Indeed major breakthroughs on the identification of genes involved in disease resistance (**Figure 1**, Martin et al., 1993; Jones et al., 1994; Salmeron et al., 1996) and the understanding of resistance complex formation and activation have been described in tomato (recently reviewed in Ntoukakis et al., 2014; decisive papers including Tang et al., 1996; Kim et al., 2002; Xing et al., 2007; Mucyn et al., 2009; Ntoukakis et al., 2009, 2013). Recently, the tomato genome was sequenced (Tomato Genome Consortium, 2012), further strengthening its position as a model for plant–pathogen interactions.

Since the 1980s, *Arabidopsis* has been extensively studied as a model within the dicotyledonous plants; milestones are described in **Figure 1**. Somerville and Koornneef (2002) reported that by the early 2000s, over 2500 papers a year were being published describing research carried out on *Arabidopsis* and in 2013 this figure was almost 5000 (PubMed searches matching “*Arabidopsis*” or “thaliana”), demonstrating the power of focusing the effort of the scientific community on this small flowering weed. The impressive amount of tools and resources made available to the plant research community has contributed to many major breakthroughs in our understanding of how a plant functions, the most striking example being the dissection of floral morphogenesis (Koornneef and Meinke, 2010). From the early days of research on *Arabidopsis*, its potential as a model for plant–pathogen interactions was recognized (Sijmons et al., 1991; Mauch-Mani and Slusarenko, 1993; Crute et al., 1994; Buell, 1998). Several natural pathogens with wide host ranges can infect *Arabidopsis*, including *Xanthomonas campestris* pv. *campestris*, *Pseudomonas viridiflava,* and *Pseudomonas syringae* (Tsuji, 1992; Jakob et al., 2002). However, others such as *Hyaloperonospora arabidopsidis* only grow and reproduce on *Arabidopsis*, hence this pathosystem can be used to study host-pathogen coevolution (Holub and Beynon, 1997; Holub, 2007, 2008; Coates and Beynon, 2010). Another interesting pathogen of *Arabidopsis* is the white rust causal agent *Albugo* (Holub et al., 1995; Thines et al., 2009) which can also cause major diseases in *Brassica* crops (Pound and Williams, 1963; Verma et al., 1975; Liu et al., 1989) and can suppress several broad-spectrum disease resistances (Cooper et al., 2008). Surprisingly, one of the most extensively studied pathogens of *Arabidopsis* is the bacterium *Pseudomonas syringae* pv. *tomato* DC3000, even though never isolated from *Arabidopsis* itself in the wild, it can perfectly proliferate and cause disease symptoms (Whalen et al., 1991; Xin and He, 2013).

## BREEDING FOR RESISTANCE: STRATEGIES DEPLOYED SO FAR

Resistant crop varieties have been selected by traditional breeding for more than 100 years, the first mention of wheat disease resistance breeding programs dating from Biffen (1905). However, at the time, traditional breeding programs were identifying and introgressing resistance sources in crops by crossing and selecting for traits well before understanding the mechanism of action of resistance (*R*) genes (refer to **Box 1** for definition of core concepts of plant immunity). Our initial understanding of plant disease resistance was shaped by the work of Flor (1955) over half a century ago who genetically defined the ‘gene-for-gene’ concept as requiring an avirulence (*Avr*) gene in the pathogen and an *R* gene in the host plant. While the first *Avr* gene (*AvrA*) was cloned 30 years ago (Staskawicz et al., 1984), the identification and cloning of single resistance loci of *R* genes took much longer. The first gene contributing to resistance, *Pto*, came from tomato and conferred resistance to *Pseudomonas syringae* strains carrying AvrPto and AvrPtoB (Martin et al., 1993; Scofield et al., 1996; Tang et al., 1996; Kim et al., 2002). Concurrently, the first ‘true’ (*nucleotide-binding site leucine-rich repeat; NLR*) *R* genes were identified in *Arabidopsis* (Bent et al., 1994; Mindrinos et al., 1994) and soon after the interacting *R* gene of Pto (*Prf*) was identified in tomato (Salmeron et al., 1996). Subsequently many other *R* genes were quickly identified, mostly from *Arabidopsis* and tomato and bestowing resistance to a variety of pathogens including viruses (Kohm et al., 1993; Whitham et al., 1994), bacteria (Bent et al., 1994; Mindrinos et al., 1994; Grant et al., 1995), fungi (Jones et al., 1994; Dixon et al., 1996), and oomycete pathogens (Botella et al., 1998). These major discoveries instigated the basis of the plant–microbe interaction field as we know it today.

**Box 1. Today’s core concepts of plant immunity.**

**Pathogen-associated molecular patterns (PAMPs)** are chemically conserved structures amongst many organisms, such as flagellin or chitin.
**PRR-triggered immunity (PTI)** relies on the activation of membrane bound pattern-recognition receptors (PRRs) upon recognition of PAMPs. PRRs are often receptor-like kinases (RLKs) themselves or associated with RLKs and activation leads to induction of signaling cascades, transcriptional reprograming and a complex defense output including the production of anti-microbial compounds. The most studied PRR is the receptor for bacterial flagellin; FLS2. PTI is considered as a basal source of resistance because it is triggered in both susceptible and resistant interactions.
**Effector/avirulence (*Avr*) genes** (often referred to as effectors) encode molecules delivered from the pathogen to the host plant. Generally effectors manipulate host immunity, thus preventing PTI from halting successful growth and reproduction of the pathogen.
**Resistance (*R*) genes** encode cytoplasmically located resistance proteins (R), also called nucleotide-binding site leucine-rich repeat (NLR) proteins.
**Effector-triggered immunity (ETI)** or *R* gene mediated resistance relies on the activation of resistance proteins in the presence of the corresponding Avr protein. This very specific R-Avr interaction is known as gene-for-gene resistance (Flor, 1971; Dangl and Jones, 2001) and occurs in some genotypes within a plant species. ETI often results in programmed cell death termed the hypersensitive response. Two well-described examples of R-Avr gene pairs come from research on *Arabidopsis* and tomato; the effector protein AvrRpm1 is recognized by Rpm1 via another host protein RIN4, AvrPto is recognized by Prf via Pto (Mackey et al., 2002; Mucyn et al., 2006).

In 2003, the characterization of the *Arabidopsis* resistance gene complement (NLRome) consisting of 149 *NLRs* contributed significantly to our understanding of *R* gene structures, both through their conservation and diversity and facilitated targeted approaches to indict specific genes in resistance (Meyers et al., 2003). Following this major development in *Arabidopsis*, which proved to be an extremely useful tool, NLRomes were investigated in more complex plant species. Thanks to the recent release of the tomato genome, a comprehensive tomato NLRome comprising 355 *NLRs* is now available (Tomato Genome Consortium, 2012; Andolfo et al., 2013, 2014; Jupe et al., 2013a). The availability of such NLRomes can really speed up the mapping and cloning of new resistance loci in segregating populations by integrating genetics and genomics; once a rough mapping position has been identified, one can pinpoint the locus to a few candidate *NLRs*.

Historically breeders have been selecting for more resistant crops by classically identifying new resistance sources and introgressing them into economically important crops. Transgenic approaches, however, are estimated to save 15 years on classical breeding for resistance (Haverkort et al., 2008), so it is essential that a new generation of modified plants comes to light. Since 1993, researchers have used genetic engineering to introduce genes of interest to existing high-yielding varieties without undesired pleiotropic effects. Up to now a few transgenic *R* genes have been transferred and tested into crops, whether coming from the same species or from wild relative species (reviewed in Dangl et al., 2013).

Deploying single *R* genes into the field has proven to be a rather transient solution to disease, for instance the *Brassica Rlm1*-mediated resistance was defeated within 5 years of deployment (Sprague et al., 2006). In order to tackle field resistance failures, several options are being explored. Previously, one strategy used to reduce the selective pressure on a pathogen to overcome resistance in the field is the use of multiline varieties. These contain multiple seed lines differing in their gene-specific resistances and therefore reduce the disease inoculum compared to a susceptible monoculture (Johnson and Allen, 1975). Another strategy, called *R* gene stacking or pyramiding, relies on the deployment of multiple *NLRs* at once in a single cultivar. Combining several *R* genes ensures that if the pathogen mutates to overcome one *R* gene, other resistance sources will continue to be effective. Such an approach has been particularly successful in potato cultivars (Kim et al., 2012; Jo et al., 2014). The critical need for new sources of resistance led researchers to look for resistance genes from wild relatives of crop species. *Rpi-vnt1.1* was transferred from *Solanum venturii* (wild potato) into *Solanum tuberosum* (edible potato) with success as tested in field trials for three consecutive years (Jones et al., 2014). *R* genes have been transferred between unrelated species with a relatively good success rate, proving to be a valuable tool to achieve durable disease resistance (Zhao et al., 2004, 2005; Yang et al., 2013a).

The obvious question is now how to extend the life of *R* genes, and for this we need to utilize models. The conservation of the signaling occurring downstream of *NLRs* across plant lineages has become evident (Maekawa et al., 2012), which is why model plants are required for detailed understanding of the mechanism underlying *R* gene activation in order to extend the life of single *R* genes. For example, the tomato *Ve1* gene was transferred successfully to *Arabidopsis* in order to dissect the signaling component involved in *Ve1*-medited resistance (Fradin et al., 2009, 2011). Similarly, despite having first identified *Rpm1/Rpg1*-mediated resistance in soybean (Staskawicz et al., 1984; Innes et al., 1993; Ashfield et al., 1995, 1998), it was further and largely characterized in *Arabidopsis*. This led to the identification of RIN4, a major negative regulator of plant defense and a target of several effector proteins (Mackey et al., 2002, 2003; Axtell and Staskawicz, 2003). Accessory proteins, such as RIN4, act as a bridge between R proteins and effectors, either as a direct virulence target of the effector (Van Der Biezen and Jones, 1998) or as a structural mimic of one [decoy (van der Hoorn and Kamoun, 2008) or bait model (Collier and Moffett, 2009)]. Subsequently, knowledge acquired in *Arabidopsis* is now being transferred back to soybean in an effort to understand the role of accessory proteins in *R* gene-mediated resistance in crop plants (Selote and Kachroo, 2010).

In some cases, *NLRs* function in pairs to mediate recognition (Eitas and Dangl, 2010) and *R* gene dimerization has been described in both model plants and crop species (Mestre and Baulcombe, 2006; Bernoux et al., 2011; Maekawa et al., 2011). For example, the role of oligomerization in effector recognition has extensively been described for the Pto/Prf interaction (Gutierrez et al., 2010; Ntoukakis et al., 2013). The use of model plants appears crucial in the discovery of the resistance complexes, and raises the importance of the transfer of the partners of the *R* genes into crops in order to recreate a functional module. For example, one *R* gene pair in *Arabidopsis* is the *RPS4*/*RRS1* pair which forms a complex *in vivo* that is able to recognize at least two bacterial effectors and a fungal effector (Birker et al., 2009; Narusaka et al., 2009; Williams et al., 2014). This *R* gene cooperation can provide disease resistance with extended spectrum as it is efficient against different classes of pathogens and has been deployed in several crops (Narusaka et al., 2013a,b, 2014; **Table 1**).

One way to tackle the durability issue of *R* genes is to study *R* gene evolution in the context of the whole resistance complex. Well-studied resistance mechanisms in tomato allowed this issue to be elegantly addressed by Grzeskowiak et al. (2014) for the Pto/Fen/Prf resistance complex. *Pto* homologs and *Prf* are co-localized genomically, suggesting that they evolved concurrently. As described earlier, R protein activation relies on a change of conformation upon perception of an effector or an effector-modified plant protein and the proper oligomerization and interaction with other proteins (Bonardi et al., 2012). Recent studies of artificial evolution of the potato resistance gene *Rx* (that confers resistance to potato virus X) showed that by mutating specific residues/regions, it is possible to manipulate the defense activation output and generate resistance to other pathogens, in this case to poplar mosaic virus (Farnham and Baulcombe, 2006; Harris et al., 2013).

## THE POWER OF MODELS: CHARACTERIZATION OF MUTANTS

*Arabidopsis* mutants have been indispensible in elucidating gene regulatory mechanisms and are a stellar example of how using a model system such as *Arabidopsis* aids understanding of how crop plants respond to biotic and abiotic stress. The well-annotated genome and the availability of mutants generated both by EMS mutagenesis (Rédei and Koncz, 1992; Greene et al., 2003) and T-DNA insertion (Alonso et al., 2003) allows rapid characterization of gene function in *Arabidopsis* using both forward and reverse genetic approaches. In the early days of molecular plant pathology, characterization of *Arabidopsis* mutants such as *non-expresser of PR genes* (*npr1*; Cao et al., 1994) and several *enhanced disease susceptibility* (*eds*) mutants (Glazebrook et al., 1996; Parker et al., 1996) allowed dissection of the genetic basis of the plant immune response for the first time. More recently, mutant screens also identified *flagellin sensing 2 (fls2)*, the most studied pattern recognition receptor (PRR) to date (Gómez-Gómez and Boller, 2000) but also one of the major regulator of PRR activation *BAK1 (brassinosteroid insensitive 1 – associated protein kinase)*, even though originally identified as a suppressor of brassinosteroid signaling (Li et al., 2002; Chinchilla et al., 2007; Heese et al., 2007); furthering our understanding of early signaling during PRR-triggered immunity (PTI; **Box 1**).

Model plants have enabled our understanding of the interplay between immunity and development (Tian et al., 2003; Heidel et al., 2004; Bolton, 2009; Göhre et al., 2012; Lozano-Durán et al., 2013). Auto-activated or constitutively expressed *NLRs* such as *SNC1* lead to dwarf phenotypes (Zhu et al., 2010), which emphasize the importance of fine-tuning of *R* gene expression regulation. Similarly the *MAP Kinase 4* (*mpk4*) mutant that exhibits constitutive activation of systemic acquired resistance, displays a dwarf phenotype (Petersen et al., 2000).

The regulation of stress responses and hormone signaling has also been elucidated using *Arabidopsis* mutants (Koornneef et al., 1984; Cao et al., 1994; Finkelstein, 1994; Xie et al., 1998). Now we have been able to characterize the antagonism in hormone signaling between salicylic acid (SA) and jasmonic acid (JA), which allows plants to tailor their defense depending on the pathogen (Glazebrook, 2005; Robert-Seilaniantz et al., 2011) and is open to manipulation by pathogens to aid colonization (Marumo et al., 1982; Valls et al., 2006; El Oirdi et al., 2011). In addition to characterization of hormone signaling pathways, we are starting to unravel their regulation. For example the *topless* developmental mutant was identified in *Arabidopsis* following EMS mutagenesis by Long et al. (2002). It has since been shown in *Arabidopsis* that in addition to regulation of auxin signaling (Osmont and Hardtke, 2008), TOPLESS (TPL) negatively regulates JA-responsive genes (Pauwels et al., 2010) and has been implicated in negative regulation of SA signaling (Arabidopsis Interactome Mapping Consortium, 2011). Recently, TPL and multiple family members have been identified in many higher plants and their localization and interaction with repressors in auxin signaling shown in tomato (Hao et al., 2014). Together with our knowledge from *Arabidopsis*, this gives potential for manipulation of hormonal defense in crop species.

The detailed analysis of mutants in *Arabidopsis* has resulted in a detailed understanding of stress signaling which is being transferred to crops. Among many other examples described is the SnRK2 family of protein kinases which play a role in abscisic acid signaling and were identified and characterized in *Arabidopsis* over a decade ago (Yoshida et al., 2002; Hrabak et al., 2003). This family is now being characterized in crop species including maize (Huai et al., 2008), sorghum (Li et al., 2010), and rice (Xu et al., 2013). Another example is the NAC family of transcription factors, which play a role in response to biotic and abiotic stress (Wang et al., 2009; Nakashima et al., 2012). Recently these NAC transcription factors have been characterized in potato, aided by the previous description of their functionality in *Arabidopsis* (Tian et al., 2003; Singh et al., 2013) and their manipulation by effectors from *Phytophthora infestans* has been shown (McLellan et al., 2013).

### PATTERN RECOGNITION: DEFENSE AGAINST CONSERVED MICROBIAL COMPONENTS

Understanding of the immune response gained from models and particularly the scrutiny of mutants has enabled us to characterize the receptors and signaling associated with plant immunity, particularly differentiating between PTI and effector-triggered immunity (ETI; **Box 1**). Using this knowledge, one way to achieve broader spectrum resistance is to make use of *PRR*s. Lacombe et al. (2010) elegantly showed that EF-Tu receptor (*EFR*), a *PRR* gene restricted to the Brassicaceae genus, confers increased resistance to various bacterial pathogens when expressed in Solanaceae. Given that *EFR* confers broad-spectrum resistance to a variety of bacterial pathogens, it is being currently tested for its action in potato, lettuce, citrus and apple^1^. This is the first example of transferring a *PRR* between genera and suggests that the downstream PAMP-induced signaling cascade is conserved between species, paving the way to further utilize this source of resistance. Similarly, *Xa21*, a *PRR* restricted to rice, was transferred to orange, tomato and banana (Ronald et al., 1992; Afroz et al., 2010; Mendes et al., 2010; Tripathi et al., 2014) and *DORN1* (*does not respond to nucleotides 1*, also called *LecRK-I.9*), the extracellular ATP (eATP) receptor, was transferred into potato (Bouwmeester et al., 2014). Even though effectors can target *PRRs* and suppress PTI outputs (Göhre et al., 2008; Gimenez-Ibanez et al., 2009; Zhang et al., 2010), *PRRs* still stand as a potent additional source of resistance for disease control. Similar to the identification of NLRomes, efforts have been made to identify PRRomes (Fritz-Laylin et al., 2005; Tang et al., 2010; Andolfo et al., 2013). Plant genomes encode hundreds of receptor-like proteins and receptor-like kinases that may be potential *PRRs* involved in disease resistance. A future promising approach to improve disease resistance could be to combine known *PRRs* and *NLR R* genes in a same cultivar that would confer simultaneously increased resistance to a wide-range of pathogens and strong resistance to specific pathogenic strains.

### NON-HOST RESISTANCE: A SOURCE OF DURABLE RESISTANCE?

Non-host resistance (NHR) arises when an entire plant species is resistant to a pathogen (Heath, 1981; Singh et al., 2013) and since it is much more durable than *R* gene mediated resistance, potentially, it could be an exciting new means of crop improvement. *Arabidopsis* displays NHR to several commercially important pathogens such as the potato blight pathogen *Phytophthora infestans* and the causal agent of Asian soybean rust (ASR), *Phakopsora pachyrhizi* (Huitema et al., 2003; Loehrer et al., 2008). It has therefore been possible to use a series of *Arabidopsis* mutants to identify components of NHR. These mutants, designated the *penetration* (*pen*) mutants, allow the penetration of fungal pathogens to which *Arabidopsis* is a non-host (Collins et al., 2003; Assaad et al., 2004). They have allowed important components of NHR to be unraveled such as PEN1 which is a membrane-anchored syntaxin involved with vesicle trafficking to the cell membrane and PEN2 and PEN3 which are thought to work together in producing and delivering antifungal compounds to the apoplast upon attempted infection (Collins et al., 2003; Lipka et al., 2005; Stein et al., 2006; Loehrer et al., 2008). Transcriptional profiling of *pen* mutants has allowed the identification of post-invasion induced NHR genes (PINGs) such as *bright trichomes 1* (*BRT1*; Langenbach et al., 2013). Critically, it has recently been shown that transferring *BRT1* from *Arabidopsis* to soybean results in reduction in disease symptoms of ASR (patent by Conrath et al., 2013).

Multiple components contribute to NHR but the exact mechanisms are not yet fully understood. NHR may partially be mediated by *R* genes, as when effectors are expressed ectopically in a non-host plant some can trigger an ETI like resistance response (Kobayashi et al., 1989). Two *Arabidopsis R* genes seem to play a role against NHR to the *Brassica* fungal pathogen *Leptosphaeria maculans* (Staal et al., 2006). Additionally, NHR in *Arabidopsis* against powdery mildew involves cell wall appositions and production of antimicrobial compounds. A proposed model by Schulze-Lefert and Panstruga (2011) suggests that the relative contribution of PTI (as opposed to recognition by R proteins) increases with evolutionary distance from the host plant. It is therefore thought that through studying NHR we may be able to find and engineer durable resistance mechanisms independently of *R* protein recognition.

## OMICS’ APPROACHES

### EFFECTORS, EFFECTOROMICS, AND PATHOGENOMICS

Pathogens deploy an arsenal of toxins and effector proteins, which play an important role in manipulation and suppression of plant defenses during disease (Jones and Dangl, 2006). Pathogen effector repertoires can vary in size; *Pseudomonas syringae* has a typical core of 10–40 effectors (Guttman et al., 2002; Petnicki-Ocwieja et al., 2002; Baltrus et al., 2011; O’Brien et al., 2011; Lindeberg et al., 2012), whereas the oomycete pathogen *Phytophthora infestans* has around 500 effectors (Haas et al., 2009; Bozkurt et al., 2012). Yeast-two-hybrid (Y2H) systems have been instrumental for the pairing of resistance proteins and effector proteins. Following the identification of Pto in tomato (Martin et al., 1993), the bacterial effectors AvrPto and AvrPtoB were identified as its interacting partners using a Y2H system (Scofield et al., 1996; Kim et al., 2002). Despite the early success of the Y2H system, subsequent experiments trying to pair R proteins and effectors had limited success. In hindsight, this is because direct interaction between R proteins and effectors appears to be the exception (Jia et al., 2000; Deslandes et al., 2003; Dodds et al., 2006; Krasileva et al., 2010; Cesari et al., 2013), often relying on accessory proteins that can bridge the two. More recent -omics studies support the idea that direct interaction is the exception and indirect interaction of the effectors with the R proteins is commonly the case (Mukhtar et al., 2011). However, effectors have still assisted us in selecting *R* genes for breeding; this was elegantly shown using the *AvrBs2* effector which is highly conserved in *Xanthomonas* species. Transfer of the corresponding resistance gene (*Bs2*) from pepper into tomato was successful at providing resistance to *Xanthomonas perforans* (Horvath et al., 2012).

Effectors can also be used as a powerful tool to dissect the plant immune response by identifying key components or modifications required for activation of immunity. A comprehensive roadmap for effector discovery and functional analysis has been described before (Alfano, 2009) with the eventual aim of engineering plants with durable disease resistance. Basic components of the roadmap are the identification of host interacting proteins, mode of function, and localization of the effector that can be valuable tools in our efforts to unravel a plant defense mechanisms. For instance, the characterization of the effector HopU1 unraveled the role of the RNA-binding protein GRP7 in immunity (Nicaise et al., 2013) and more recently Macho et al. (2014) benefited from the functional characterization the effector HopAO1to dissect the role of tyrosine phosphorylation in PTI.

Using model systems has facilitated Y2H genome-wide studies which have resulted in the identification of *Arabidopsis* protein ‘hubs’ that are converged on by independently evolved effectors from multiple species (Mukhtar et al., 2011; Weßling et al., 2014). It is also possible to tie this immune network into a larger *Arabidopsis* interaction network (Arabidopsis Interactome Mapping Consortium, 2011) and it has been observed that hubs targeted by effectors from multiple pathogens appear to be more likely to have a role in immunity. The next question naturally is therefore whether one can observe similar hubs in crops.

### TRANSCRIPTOMICS

The Arabidopsis community has been greatly aided by the availability of post-genomic resources such as The Arabidopsis Information Resource^2^ (Rhee, 2003) and the Arabidopsis Information Portal^3^, lab based tools such as whole genome microarrays (Aharoni and Vorst, 2002; Redman et al., 2004) and web tools such as ‘genevestigator’ (Zimmermann et al., 2004) and the ‘Electronic Fluorescent Pictograph’ browser (Winter et al., 2007) for exploring large transcriptomic data sets and gene expression in different contexts. All of these resources have aided annotation of crop genomes, for example it was only in 2011 that the 1.2 Gb oilseed rape genome was dissected using transcriptome sequencing and the *Arabidopsis* genome as a reference for alignment (Bancroft et al., 2011).

One of the great benefits of these resources in models is the ability to perform in-depth studies of plant–pathogen interactions at the transcriptional level. For example, Thilmony et al. (2006) started to unravel manipulation of host transcription during *Arabidopsis* infection with the infectious *Pseudomonas syringae* strain DC3000 in addition to a *Pseudomonas syringae hrpA* mutant (compromised in its ability to deliver type III effector proteins (Collmer et al., 2000) and the *Pseudomonas syringae* DC3118 COR- mutant (defective in coronatine toxin production). This allowed them to begin separating host-mediated transcriptional changes from those caused by the action of pathogen effectors and virulence factors. Such studies are key to identifying ‘desirable’ transcriptional responses from the point of view of the host plant that can be used in future crop improvement applications. Models have also allowed detailed infection time courses to be performed, for example during *Phytophthora capsici* infection of tomato (Jupe et al., 2013b) and during *Botrytis cinerea* infection of *Arabidopsis* (Windram et al., 2012). These studies have given a detailed analysis of transcriptional reprogramming during colonization by a hemi-biotrophic and necrotrophic pathogen respectively. The beauty of such approaches is that in addition to providing high-resolution transcriptional information that has facilitated the development of a plethora of bioinformatic tools (Brown et al., 2013; Polanski et al., 2014), it provides detailed insights into the chronology of infection, allowing clustering of similarly expressed genes. This in turn can be used for subsequent generation of network inference models (Penfold and Wild, 2011; Penfold et al., 2012). Such models enable the development of testable hypotheses as to the role and significance of specific genes in regulatory networks induced during biotic stress.

In addition, some studies are starting to link gene regulatory networks in *Arabidopsis* with that of crops. For example, by using conserved non-coding sequences it was possible to identify shared components of regulatory networks in *Arabidopsis*, papaya, poplar, and grape (Baxter et al., 2012). Recently, so-called transcription hubs have been identified either as targets of multiple effectors or transcriptional hubs regulating responses to pathogens (Windram et al., 2014) which give us potential targets for manipulation by new genome editing and synthetic biology technologies.

## LOOKING TO THE FUTURE: NEW TECHNOLOGIES FOR CROP IMPROVEMENT

Through studying model plants and their pathogens, we have identified both protein and transcriptional hubs in plant immunity, which give us obvious targets for manipulation in crops. The plant community has two very promising tools to exploit for genome editing; the first of which is the transcription activator-like effector nuclease system (TALEN; Christian et al., 2010; Bogdanove and Voytas, 2011; Schornack et al., 2013) which utilizes the ability of TAL effectors from plant–pathogenic *Xanthomonas* species to bind short regions of DNA in a sequence specific manner (Boch et al., 2009) and modulate gene expression. The second tool is the clustered regulatory interspaced short palindromic repeat (CRISPR) system (Belhaj et al., 2013). This technology gives us the ability to create point mutations in genes without inserting extra unnecessary foreigner DNA. For example homozygous targeted gene knock-out plants were obtained at the first generation in tomato (Brooks et al., 2014) and is particularly important due to the skepticism of GM crops generally exhibited by the public.

In the last few years, many tools have been developed which allow the rapid cloning and assembly of modular constructs that would have been previously challenging. For example USER fusion, which utilizes uracil excision-based cloning (Geu-Flores et al., 2007), Golden Gate and golden gate-based systems such as golden braid (Engler et al., 2008; Sarrion-Perdigones et al., 2013). There are also molecular toolkits available (Binder et al., 2014; Engler et al., 2014) that allow rapid and flexible cloning. These technologies can be used both to complement existing *R* gene mediated immunity by stacking *R* genes in so-called resistance cassettes (multi-*R-*gene constructs), and in new synthetic biology approaches. Synthetic biology approaches have the exciting potential for designing inducible disease resistance in crops; it has been shown to be possible to engineer synthetic, stress responsive promoters in *Arabidopsis* (Hou et al., 2012). Our detailed knowledge of signaling in plant immunity, resulting form genetics, mutations, genomics and systems modeling will enable the construction of novel, resilient immune response networks in plants.

Synthetic biology can also be combined with existing tools; such as utilizing the DNA binding properties of TAL effectors for more than nuclease targeting. For example, since it is known that expression of the rice resistance gene *Xa27* is mediated through the presence of a TAL effector binding site for the effector *avrXa27* (Gu et al., 2005), there is potential for engineering ‘designer *R* genes.’ By using the TAL effector code there is potential to design several binding sites and engineer an *R* gene that mounts resistance to several conserved pathogen effectors (reviewed in Grant et al., 2013).

These new techniques can help to tackle major challenges associated with crops, including polyploidy genomes (such as strawberries Galletta and Maas, 1990). For example TAL effector and CRISPR-based technology was used to successfully mutate the three homeoalleles of the powdery mildew susceptibility gene *Mlo* from the hexaploid wheat in order to prevent pathogen growth (Wang et al., 2014). In addition, we need to design plants that are able to respond to pathogen attack without developmental penalties and this looks to be achievable using synthetic biology approaches.

## CONCLUSION

The use of plant models such as *Arabidopsis* and tomato has been instrumental in addressing the mechanisms of plant–microbe interactions. Models have especially helped formulate concepts to describe the plant immune system (Jones and Dangl, 2006). Due to the wide-range of pathogens able to infect these two model plants, and their different modes of infection, the scientific community has been able to dissect at the molecular level the dialog established between the host and its pathogen and comprehend what lies behind plant disease susceptibility and plant disease resistance. The elucidation of how PRRs and R proteins are activated and trigger downstream signaling to fulfill resistance has highlighted many key players to target for increased resistance in crop plants. However, overall knowledge transfer from basic research to crop plants has been rather limited (**Table 1**). There are not many examples that have made it to the level of commercial production and progress here has been disappointingly slow considering how much we have learned about plant innate immunity in recent years. The characterization of defense gene regulatory networks in model plants will be the next step that will expedites the transposition of knowledge into crops. The new tools of synthetic biology approaches will further enable plant breeders to engineer designer crops with inducible defenses against pathogens lacking the corresponding fitness costs that may make them unappealing to growers. The future challenge for plant pathology will be to leverage this increasing knowledge base in the models to engineer durable resistance in the major crop plants to sustain yield in the face of altering climatic conditions.

## Conflict of Interest Statement

The authors declare that the research was conducted in the absence of any commercial or financial relationships that could be construed as a potential conflict of interest.

## Footnotes

1 http://2blades.org

2 http://www.arabidopsis.org

3 https://www.araport.org
